# Fast Time Synchronization on Tens of Picoseconds Level Using Uncombined GNSS Carrier Phase of Zero/Short Baseline

**DOI:** 10.3390/s20174882

**Published:** 2020-08-28

**Authors:** Yinghao Zhao, Letao Zhou, Wei Feng, Shaoguang Xu

**Affiliations:** Faculty of Geosciences and Environmental Engineering, Southwest Jiaotong University, Chengdu 611756, China; justdoitzyh@my.swjtu.edu.cn (Y.Z.); wfeng@swjtu.edu.cn (W.F.); shaoguangxu@home.swjtu.edu.cn (S.X.)

**Keywords:** GNSS carrier phase, time synchronization, tens of picoseconds level, between-receiver IFB, single-difference ambiguity resolution, zero/short baseline

## Abstract

Since the observation precision of the Global Navigation Satellite System (GNSS) carrier phase is on the order of millimeters, if the phase ambiguity is correctly solved, while calibrating the receiver inter-frequency bias, time synchronization on the order of tens of picoseconds is expected. In this contribution, a method that considers the prior constraints of the between-receiver inter-frequency bias (IFB) and its random variation characteristics is proposed for the estimation of the between-receiver clock difference, based on the uncombined GNSS carrier phase and pseudorange observations of the zero and short baselines. The proposed method can rapidly achieve the single-difference ambiguity resolution of the zero and short baselines, and then obtain the high-precision relative clock offset, by using only the carrier phase observations, along with the between-receiver IFBs being simultaneously determined. Our numerical tests, carried out using GNSS observations sampled every 30 s by a dedicatedly selected set of zero and short baselines, show that the method can fix the between-receiver single-difference ambiguity successfully within an average of fewer than 2 epochs (interval 30 s). Then, a clock difference between two receivers with millimeter precision is obtained, achieving time synchronization on tens of picoseconds level, and deriving a frequency stability of 5 × 10^−14^ for averaging times of 30,000 s. Furthermore, the proposed approach is compared with the precise point positioning (PPP) time transfer method. The results show that, for different types of receivers, the agreement between the two methods is between −6.7 ns and 0.2 ns.

## 1. Introduction

Time synchronization plays an important role in our daily life. In particular, communication systems, power grids and financial networks all rely on precise timing to achieve synchronization and operation efficiency. Furthermore, in the maintenance of the spatial-temporal reference frame, the precision of time synchronization is also crucial. In the early 1980s, when the radio signals from the satellites of the Global Positioning System (GPS) began to be used for timing services, time keeping changed dramatically [1]. So far, time and frequency transfer based on Global Navigation Satellite System (GNSS) technology has made great progress. According to [2,3], GNSS time transfer technology was clarified into three groups, i.e., the common view (CV) method, all in view (AV) method, and carrier phase (CP) method. Both the CV and AV methods are based on GNSS code pseudorange [4,5,6]. In case of GNSS pseudorange, time and frequency transfer is not limited to the distance constraints; as well as the data processing being simple and easy to operate, and it has been widely used for the timing service. However, the accuracy is recognized to be limited, and the theoretical precision of the receiver clock offset, as determined by pseudorange observations (noise level 30 cm), can reach 1 ns at most. Additionally, it is generally known that the day-boundary jumps are due to the long-term pseudorange noise [7,8]. Fortunately, the precision of GNSS carrier phase observation can be less than 1 mm [9]. Compared with the precision of pseudorange, the precision of carrier phase is improved by two orders of magnitude, which is of great significance for the high-precision timing of real-time applications.

Based on the observation model, that is, whether to make a difference between the observations of the two stations, the CP time transfer method can be divided into two types: the zero-difference (ZD) method and the single-difference (SD) method. The ZD method is mainly based on the precise point positioning (PPP) technology, to estimate the receiver clock offset, which does not require any observation synchronization. This method is flexible, convenient, and highly accurate [10,11,12]. Conventionally, the ionospheric-free code and phase combinations are employed for time and frequency transfer [13,14,15]. This makes it hard to solve the combined phase ambiguity [16], and the estimated receiver clock offset is easily affected by the combined measurement noise and receiver hardware delay [17]. In addition, high-precision satellite orbit and clock products are crucial for the precision and efficiency of this method [18]. Since there is a need for the accurate correction of various errors, it is difficult to determine the receiver clock offset quickly and accurately in a short period of time, and hard to meet the high timeliness requirements of high-precision timing applications. By making a difference between the observations of two stations, the satellite-dependent errors, such as satellite clock error and satellite hardware bias, can be completely eliminated. Meanwhile, the space-related errors are weakened to a certain extent, leading to the fast determination of relative clock offset. The estimation of between-receiver clock difference using the single-differenced GPS ionosphere-free code and phase combinations has been investigated [19]. Their numerical results indicated that agreement with the two-way satellite time and frequency transfer (TWSTFT) at the level of 0.3 nanoseconds could be achieved even on transatlantic baselines. However, the estimated between-receiver clock difference was affected by the combined observation noise, and the ambiguity resolution was complicated. If the uncombined GNSS carrier phase observations are utilized to estimate the receiver clock difference, the results are expected to be less affected by the measurement noise and other errors. It is of great significance for the application of tens of picoseconds level time synchronization.

To summarize, using the carrier phase observation for time synchronization can achieve higher precision, but there are generally problems of poor timeliness due to the difficulty of solving the phase ambiguity, and easily being affected by the receiver hardware delay. In order to improve the precision and efficiency of the receiver clock offset estimation, two key issues need to be resolved: one is to deal with the receiver hardware bias reasonably, and the other is to fix the phase integer ambiguity rapidly and successfully. In this study, we assessed the precision and efficiency of the CP time-frequency synchronization using the uncombined GNSS carrier phase observations, considering the prior constraints of the between-receiver inter-frequency biases (IFBs). The mathematical principle and the data processing strategies for estimating the between-receiver clock difference were addressed. First, we analyzed and modeled the temporal variation characteristics of the between-receiver IFBs. Then, a method that took the prior constraints of between-receiver IFB and its random variation characteristics into account was proposed to obtain the between-receiver clock difference, by using only the uncombined GNSS carrier phase observation, which pays special attention to the resolution of the between-receiver single-difference ambiguity.

In Section 2, we describe the principles of time synchronization using the uncombined GNSS carrier phase and pseudorange observations of a short baseline, with special attention paid to the handling of the between-receiver IFBs and the resolution of the between-receiver single-difference ambiguity. In the subsequent Section 3, we detail the experimental design and data processing strategies used to assess the performance of the proposed method. Following this, the numerical results are presented and discussed in Section 4. Finally, some conclusions are given in Section 5.

## 2. Principles of the between-Receiver Clock Difference Estimation

This section begins with the general observation models of GNSS signals. Then, the CP time synchronization model based on the uncombined carrier phase observations of the short baseline are developed in detail, which considers the prior constraints of the between-receiver IFB and its random variation characteristics. Finally, the method of parameter estimation is given.

### 2.1. Observation Equation

For a short baseline, the between-receiver single-difference equations of GNSS carrier phase and pseudorange observations can be described as [20]:

(1) $$\begin{array}{ccc} L_{ur,j}^{s}\left(t\right) & = & \rho_{ur}^{s}+\zeta_{ur,j}^{s}+cdt_{ur}+\delta_{ur,j}+\omega_{ur,j}^{s}+\lambda_{j}N_{ur,j}^{s}+\varepsilon_{ur,j}^{s}\\ P_{ur,j}^{s}\left(t\right) & = & \rho_{ur}^{s}+\zeta_{ur,j}^{s}+cdt_{ur}+d_{ur,j}+\upsilon_{ur,j}^{s} \end{array}$$

where ${\left(\cdot \right)}_{ur}^{s}={\left(\cdot \right)}_{u}^{s}-{\left(\cdot \right)}_{r}^{s}$ denotes the across-receiver difference, which is the difference of simultaneous observations taken at stations u and r to the satellite s. The subscript j denotes the corresponding frequency band, t denotes the epoch, L and P are the carrier phase and pseudorange observations, ρ is the geometric range, which refers to the satellite center of mass and the receiving antenna reference point, and ζ contains the correction due to phase center offsets of the transmitting and receiving antennas. ω is the phase wind-up correction, which accounts for a change in the measured phase for the case of the rotations of the antennas and can be accurately modeled as described in [21]. c is the light speed in the vacuum, dt is the receiver clock error, δ is the receiver phase hardware bias; d is the receiver code hardware bias, λ is the wavelength, N denotes the phase integer ambiguity, ε and υ are the phase and code measurement noise, respectively.

As is known, the spatial correlation between the observation error sources of two neighborhood stations are highly related to the length of the baseline. Since the length of baseline studied in this research is tens of meters and the heights of the two stations are basically the same, the single-difference tropospheric and ionospheric delays in (1) were ignored. Because the satellite-dependent part is assumed to be equal for every receiver tracking the corresponding signal, the terms for the satellite clock offset and the relativistic correction for the satellite clock due to noncircular orbits are dropped out of (1). Besides, for signals, e.g., BeiDou Navigation Satellite System (BDS) signals and GPS signals, that are based on code division multiple access (CDMA) technology, the frequency is identical for all channels, and the satellite hardware bias of a single signal can thus be eliminated by making a difference between two receivers [22].

Due to the receiver phase or code hardware bias of a single signal linearly corelated to the receiver clock offset, the absolute quantities of the two cannot be separated without external observation information. Thus, for the dual-frequency band (j=1, 2) receiver, the receiver phase hardware bias on the *L*_1_ carrier phase is lumped to the receiver clock offset parameter based on (1). Then, we can obtain the re-parameterized equations of the dual-frequency carrier phase and pseudorange observations, as shown below:

(2) $$\begin{array}{ccc} L_{ur,1}^{s}\left(t\right) & = & \rho_{ur}^{s}+c\tau_{ur}+\lambda_{1}N_{ur,1}^{s}+\varepsilon_{ur,1}^{s}\\ L_{ur,2}^{s}\left(t\right) & = & \rho_{ur}^{s}+c\tau_{ur}+b_{ur,L_{2}-L_{1}}+\lambda_{2}N_{ur,2}^{s}+\varepsilon_{ur,2}^{s}\\ P_{ur,1}^{s}\left(t\right) & = & \rho_{ur}^{s}+c\tau_{ur}+b_{ur,P_{1}-L_{1}}+\upsilon_{ur,1}\\ P_{ur,2}^{s}\left(t\right) & = & \rho_{ur}^{s}+c\tau_{ur}+b_{ur,P_{2}-L_{1}}+\upsilon_{ur,2} \end{array}$$

in which, the re-parameterized parameters were

(3) $$\begin{array}{ccc} c\tau_{ur} & = & cdt_{ur}+\delta_{ur,1}\\ b_{ur,L_{2}-L_{1}} & = & \delta_{ur,2}-\delta_{ur,1}\\ b_{ur,P_{1}-L_{1}} & = & d_{ur,1}-\delta_{ur,1}\\ b_{ur,P_{2}-L_{1}} & = & d_{ur,2}-\delta_{ur,1} \end{array}$$

with $c\tau_{ur}$ as the re-parameterized between-receiver clock difference, that contains the relative clock offset and the between-receiver phase hardware bias of *L*_1_ carrier phase, $b_{ur,L_{2}-L_{1}}$ is the between-receiver phase-to-phase IFB ($\Delta {\mathrm{IFB}}_{L_{2}-L_{1}}$), $b_{ur,P_{1}-L_{1}}$ and $b_{ur,P_{2}-L_{1}}$ are the between-receiver code-to-phase IFBs ($\Delta {\mathrm{IFB}}_{P_{1}-L_{1}}$ and $\Delta {\mathrm{IFB}}_{P_{2}-L_{1}}$). Here, the equations for the estimation of the interested parameters were established by a re-parameterization processing. Following this, the between-receiver IFBs should be proper handled in order to achieve the accurate and reliable estimates of between-receiver clock difference.

### 2.2. Prior Constraints and State Models of the between-Receiver IFBs

When the station coordinates were accurately known, the unknown parameters in (2) included the between-receiver clock difference, between-receiver IFBs, and between-receiver single-difference ambiguities. Among them, the receiver hardware bias is closely related to the hardware characteristics of the receiver itself. For two receivers of the same type, their correlators and font-end designs are essentially the same, so the hardware bias of the two receivers for the same observation is roughly the same. It is reasonable to assume that the mathematical expectation of the relative deviation, after differencing values from two receivers of the same type, should be close to zero. Furthermore, the existing studies of the GLONASS receiver IFB have shown that the quantity of receiver inter-frequency phase bias is in the range of centimeters to decimeters [23,24]. Even for different types of receiver pairs, it is possible to constrain the between-receiver IFBs and set a reasonable variance. Therefore, the between-receiver phase-to-phase IFB and code-to-phase IFB can be restricted as follows:

(4) $$\begin{array}{ccc} b_{ur,L_{2}-L_{1}}^{0} & = & b_{ur,L_{2}-L_{1}},\;\sigma_{b_{ur,L_{2}-L_{1}}^{0}}^{2}\\ b_{ur,P_{1}-L_{1}}^{0} & = & b_{ur,P_{1}-L_{1}},\;\sigma_{b_{ur,P_{1}-L_{1}}^{0}}^{2}\\ b_{ur,P_{2}-L_{1}}^{0} & = & b_{ur,P_{2}-L_{1}},\;\sigma_{b_{ur,P_{2}-L_{1}}^{0}}^{2} \end{array}$$

in which, the item $b_{ur,L_{2}-L_{1}}^{0}$ is the priori value of $\Delta {\mathrm{IFB}}_{L_{2}-L_{1}}$, $b_{ur,P_{1}-L_{1}}^{0}$ and $b_{ur,P_{2}-L_{1}}^{0}$ are the priori values of $\Delta {\mathrm{IFB}}_{P_{1}-L_{1}}$ and $\Delta {\mathrm{IFB}}_{P_{2}-L_{1}}$, respectively. Then, a variance ($\;\sigma_{b_{ur,L_{2}-L_{1}}^{0}}^{2}$, $\sigma_{b_{ur,P_{1}-L_{1}}^{0}}^{2}$ or $\sigma_{b_{ur,P_{2}-L_{1}}^{0}}^{2}$) was given to describe the uncertain parts of the priori constraint. According to the existing research efforts, if the priori values of the between-receiver IFBs were set as zeroes, the standard deviation of the between-receiver phase-to-phase IFB was at decimeter level, and that of the between-receiver code-to-phase IFB was at the several meters level [25].

Above, we described the establishment of the observation equations and the addition of prior constraints to the receiver IFBs, to estimate the interest parameters. After that, the state model of the estimated parameters should be determined correctly. In a short time, a linear model (estimating the velocity of the relative clock offset) can be used to describe the state change of the receiver clock error. In continuous arcs without cycle slips, the ambiguity was usually treated as a constant. However, the between-receiver phase-to-phase IFB and code-to-phase IFB, especially the state change characteristics of the latter, is relatively lacking. Therefore, it is necessary to analyze the temporal variation characteristics of the between-receiver IFBs, and then model their state change process well [26].

Usually, the researchers constructed the geometry-free phase/code combination to estimate the receiver differential phase/code bias (DPB/DCB) based on (1). Then, the variation characteristics of the receiver DCB/DPB were analyzed, and the factors that affected the variation were investigated. The retrieval of between-receiver DCB and modeling its variation characteristics have been explored using the zero and short baselines [27]. It was found that the change process of the between-receiver DCB could be described by random walk, and the empirical variance of the receiver DCB state transition noise was given. For the DPB, a strategy to obtain the biased estimation of the between-receiver DPB was developed in [28]. By using this method, the biased estimates of the between-receiver IFBs in (2) can be obtained. Their forms are written as:

(5) $$\begin{array}{ccc} {\tilde{b}}_{ur,L_{2}-L_{1}}\left(t\right) & = & b_{ur,L_{2}-L_{1}}+\lambda_{2}N_{ur,2}^{s}-\lambda_{1}N_{ur,1}^{s}\\ {\tilde{b}}_{ur,P_{1}-L_{1}}\left(t\right) & = & b_{ur,P_{1}-L_{1}}-\lambda_{1}N_{ur,1}^{s}\\ {\tilde{b}}_{ur,P_{2}-L_{1}}\left(t\right) & = & b_{ur,P_{2}-L_{1}}-\lambda_{1}N_{ur,1}^{s} \end{array}$$

They are biased because the phase ambiguities of the reference satellite in the first epoch are contained in the estimation. Since the ambiguity remains constant in continuous arcs without cycle slips, the actual epoch variation of the between-receiver IFBs can be obtained by performing time difference on the biased estimates, which read as:

(6) $$\begin{array}{ccc} \nabla b_{ur,L_{2}-L_{1}}\left(t\right) & = & {\tilde{b}}_{ur,L_{2}-L_{1}}\left(t\right)-{\tilde{b}}_{ur,L_{2}-L_{1}}\left(t-1\right)\\ \nabla b_{ur,P_{1}-L_{1}}\left(t\right) & = & {\tilde{b}}_{ur,P_{1}-L_{1}}\left(t\right)-{\tilde{b}}_{ur,P_{1}-L_{1}}\left(t-1\right)\\ \nabla b_{ur,P_{2}-L_{1}}\left(t\right) & = & {\tilde{b}}_{ur,P_{2}-L_{1}}\left(t\right)-{\tilde{b}}_{ur,P_{2}-L_{1}}\left(t-1\right) \end{array}$$

where, the terms $\nabla b_{ur,L_{2}-L_{1}}$, $\nabla b_{ur,P_{1}-L_{1}}$ and $\nabla b_{ur,P_{2}-L_{1}}$ are the epoch variations of $\Delta {\mathrm{IFB}}_{L_{2}-L_{1}}$, $\Delta {\mathrm{IFB}}_{P_{1}-L_{1}}$ and $\Delta {\mathrm{IFB}}_{P_{2}-L_{1}}$ at epoch t, respectively. Then, the obtained epoch variations of between-receiver IFBs will be statistically analyzed with reference to the analysis strategies presented in [27], and their dynamic characteristics are summarized. Following these statistical analyses, the state equations and state process noise variances of the between-receiver IFBs are determined.

### 2.3. Parameter Estimation

In conjunction with the observation equation and the state change process of the parameters to be estimated, the matrix form of the parameter estimation for Kalman filtering can be expressed as:

(7) $$\begin{array}{l} \left[\begin{array}{c} Z_{L}\\ Z_{P} \end{array}\right]=\left[\begin{array}{cc} A_{L} & \lambda \\ A_{P} & 0 \end{array}\right]\left[\begin{array}{c} X_{i}\\ N_{i} \end{array}\right]+V_{i}\;\;\;\;\;\;\;\;\;\;\;\;V_{i}\sim N(0,Q)\\ \left[\begin{array}{cc} F & 0\\ 0 & E \end{array}\right]\left[\begin{array}{c} {\hat{X}}_{i-1}\\ {\hat{N}}_{i-1} \end{array}\right]=\left[\begin{array}{c} X_{i}\\ N_{i} \end{array}\right]+W_{i}\;\;\;\;\;\;\;\;\;W_{i}\sim N(0,R) \end{array}$$

with i is the epoch number; $X={\left[\tau_{ur},\;v_{\tau_{ur}},\;b_{ur,L_{2}-L_{1}},\;b_{ur,P_{1}-L_{1}},\;b_{ur,P_{2}-L_{1}}\right]}^{T}$ is the parameter vector to be estimated, in which, the term $v_{\tau_{ur}}$ denotes the velocity of the between-receiver clock difference; $N={\left[N_{ur,1}^{s},\;N_{ur,2}^{s}\right]}^{T}$ is the vector of the between-receiver single-difference phase ambiguity; $Z_{L}$ and $Z_{P}$ are the vectors of the carrier phase and pseudorange observations, respectively; Correspondingly, $A_{L}$ and $A_{P}$ are the coefficient matrixes of the carrier phase and pseudorange observation equations; λ is a block-diagonal matrix formed by the wavelength of the carrier signal; V represents the observation noise matrix; Q is the variance matrix of the observations, and the diagonal elements are determined by the satellite elevation weighted model; $F=\left[\begin{array}{ccc} 1 & \Delta t & 0\\ 0 & 1 & 0\\ 0 & 0 & I \end{array}\right]$ is the matrix of the state transition coefficient with five rows and five columns, in which Δt is the time interval between two adjacent epochs, and I is a unit matrix with three rows and three columns; E is a unit matrix; W is the state transition process noise matrix of the estimated parameters, and the corresponding variance matrix of the state transition process noise is R. In the next section, when dealing with the measured GNSS observations, we discuss and give the specific settings of the initial state value, initial variance of the estimable parameters, and the noise variance of the state transition process.

A Kalman filter was deployed to estimate the parameters on a satellite-by-satellite and epoch-by-epoch basis. In each epoch, the float solution $\hat{N}$ and its variance-covariance matrix $Q_{N}$ were obtained. Then, the least-squares ambiguity decorrelation adjustment (LAMBDA) method was employed to search for the reduced correlation ambiguity [29], which yielded:

(8) $$\overset{⌣}{N}=\mathrm{LAMBDA}\left(\begin{array}{cc} \hat{N}, & Q_{N} \end{array}\right)$$

in which, $\overset{⌣}{N}$ is the integer option of the single-difference ambiguity. The integer solution of the ambiguity was determined according to the ratio function [30]. Once it passed the test, the integer solution of the ambiguity was substituted into the carrier phase observation equation, to recalculate the between-receiver clock difference and other parameters. The variance-covariance matrix of the fixed solution of the parameters was given in [29]. Using this formula, we calculated the root mean square (RMS) values of the fixed solution of the estimated parameters as an index to evaluate the precision of the estimation.

## 3. Experiment Data and Processing Strategy

In order to verify the precision and reliability of the proposed method, the GNSS carrier phase and pseudorange observations recorded by the zero and short baselines composed of different types of receivers were dedicatedly selected. The strategies of data processing were then described in detail.

### 3.1. Data Collection

The details of the selected stations, including the site names, receiver and antenna types, observations, and time period of the observations, were summarized in Table 1. Among them, the two stations CUT0 and CUT2 are co-located at the main campus of Curtin University in Perth, Australia. They share one antenna (TRM59800.00) to constitute a zero baseline. The last two stations YARR and YAR3, participating in the International GNSS Service (IGS) network, create a short baseline, with an approximate distance of 20.21 m. For all the cases, these receivers can acquire code and phase observations at the BDS B1 (1561.098 MHz) and B2 (1207.14 MHz) frequencies, along with GPS ones at L1 (1575.42 MHz) and L2 (1227.60 MHz) frequencies. The sampling interval of the experimental data is 30 s. The selected observation data covered a three-day period of DOY (Day of Year) 12–14 in 2018.

### 3.2. Data Processing Strategy

Table 2 summarizes the detailed GNSS data processing strategies. The between-receiver clock difference, along with the between-receiver phase-to-phase IFB, between-receiver code-to-phase IFB, and between-receiver single-difference ambiguity, were taken as the unknown parameters to be estimated, based on the estimation method described in the above section. Among them, the state change of the relative clock offset between two adjacent epochs was described by the linear model (estimating the velocity of the relative clock offset). Since existing research shows that there are short-term changes in receiver IFBs due to some factors [31], the state change of between-receiver phase-to-phase IFB and code-to-phase IFB was treated by random walk model, whereas the noise variance of the state transition process was set according to the statistical analysis in the next section. The phase ambiguity was treated as a constant in a continuous arc. The position of the satellite was calculated with the precise orbit product released by the Helmholtz-Centre Potsdam (GFZ), and the coordinates of the station under the ITRF 2014 reference frame were known. The specific processing strategies of other errors were shown in Table 2.

## 4. Results and Analysis

In this section, the numerical tests were carried out, and the experimental results were illustrated. Prior to our analysis, proper handling of the between-receiver IFBs had to be in place. Thus, we first analyzed and molded the temporal variation characteristics of the between-receiver IFBs, and then the ambiguity fixed solutions of the between-receiver clock difference and between-receiver IFBs were presented and discussed. In the end, we assessed the consistency of our proposed method with the PPP solution.

### 4.1. Temporal Variation Characteristics of the between-Receiver IFBs

In order to better constrain and describe the change process of the receiver IFBs and to set a reasonable configuration for parameter filtering, we first used the measured data to analyze and model the temporal variation characteristics of the between-receiver IFBs. The BDS carrier phase and pseudorange observations, collected from the zero baseline CUT0–CUT2 and the short baseline YARR–YAR3, were processed to obtain the biased estimation of the between-receiver IFBs, as demonstrated in (5). Since the between-receiver IFB is principally the same for all satellites of one constellation, the carrier phase and pseudorange observations of the satellite with the highest elevation angle were selected for the parameter estimation in each epoch, for the purpose of reducing the influence of the measurement noise.

**Figure 1:**
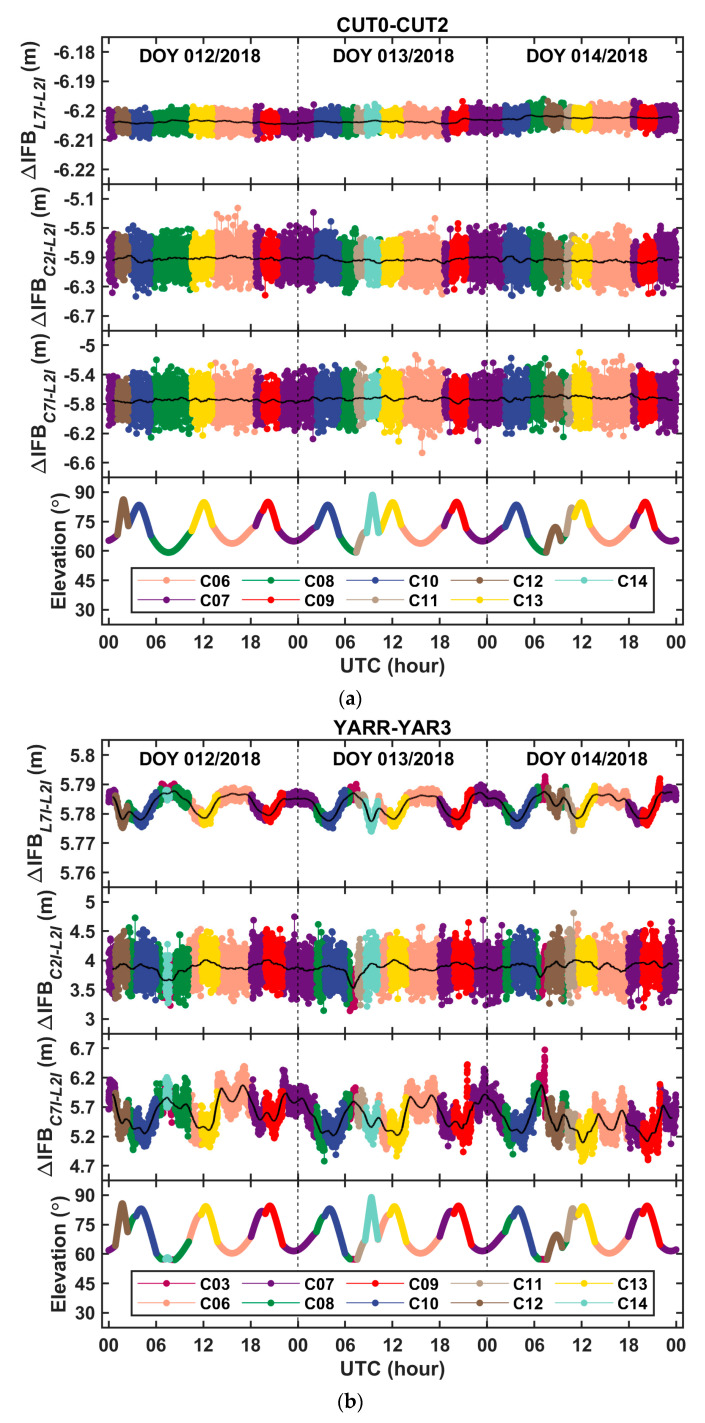
Time series of the biased estimates of between-receiver IFBs on days 12–14 in 2018 derived from (**a**) the zero baseline CUT0–CUT2 and (**b**) the short baseline YARR–YAR3.

As shown in Figure 1, the time series of the biased estimation of the between-receiver phase-to-phase and code-to-phase IFBs was obtained on days 12–14 in 2018. Obviously, the results were still contaminated by the measurement noise, so a moving average tool with a window covered 120 epochs was used to implement the denoising. The results after noise reduction are shown in the black curve in Figure 1. From Figure 1, it can be seen that the intra-day change of the between-receiver IFBs was continuous and slow. Among them, the change in the estimates of the between-receiver phase-to-phase IFB was at the centimeter level, while that of the between-receiver code-to-phase IFB was at the decimeter level. Therefore, it can be considered that, in a short period of time, the epoch variation of between-receiver IFBs is almost unchanged. Thus, the state transition coefficients of the between-receiver IFBs are reasonably set to 1. However, a reasonable state transition process noise variance still needs to be set. We addressed this issue by performing the time difference on the time series of the denoising results. The time series after noise reduction was carried out by epoch difference, to obtain the change amount of the between-receiver IFB between two adjacent epochs, and the statistical analysis was then carried out.

**Figure 2:**
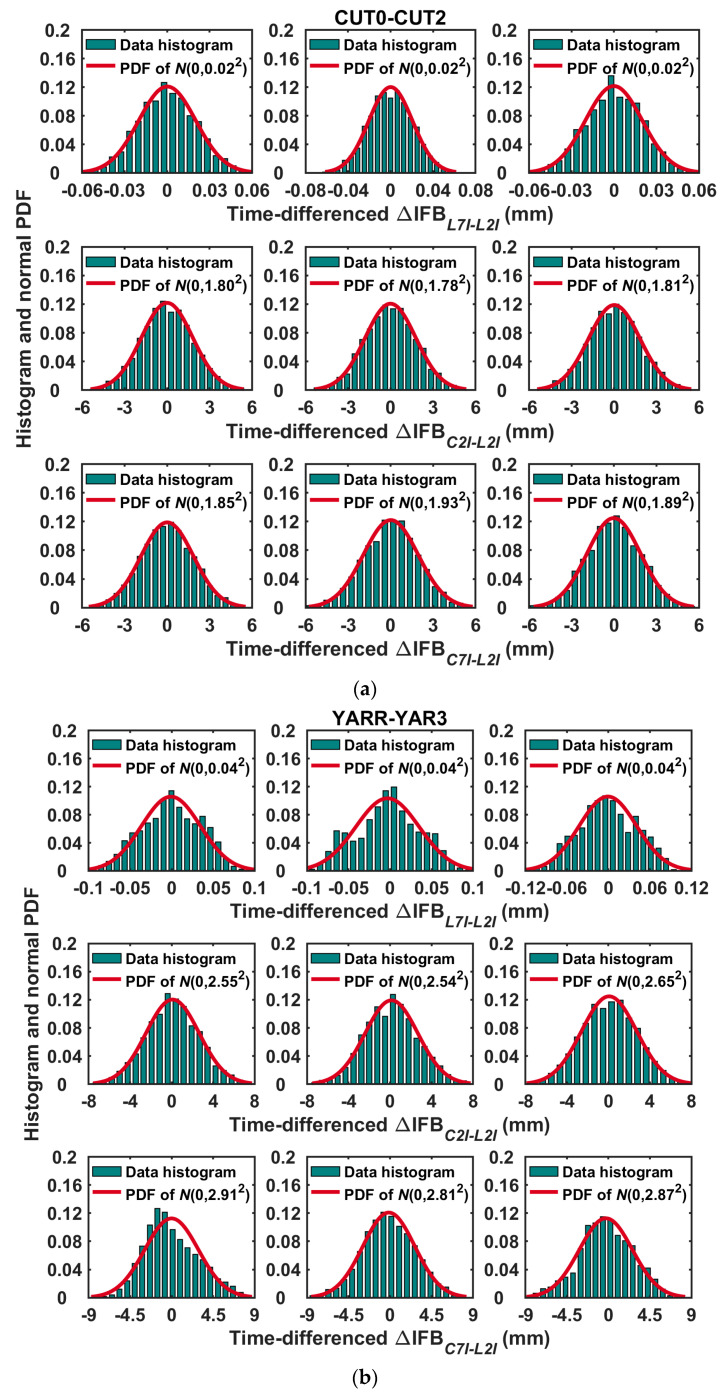
Probability distribution histogram of the epoch variation of between-receiver IFBs on days 12–14 in 2018 for (**a**) the zero baseline CUT0–CUT2 and (**b**) the short baseline YARR–YAR3.

The probability distribution histograms of epoch variation of the between-receiver IFBs were plotted in Figure 2. We can see that the epoch variation of the between-receiver IFBs approximately obeys the normal distribution. Hence, the random walk process can be used to describe the state change of the between-receiver IFBs. Table 3 shows the mathematical expectations (***e***) and standard deviations (***σ***) of the epoch-difference between-receiver IFBs of the zero baseline CUT0–CUT2 and the short baseline YARR–YAR3, on days 12–14 in 2018. The three-fold sigma (3***σ***) was expressed as a function of time, and the coefficient (***w***) of the process noise variances of the between-receiver IFBs were then obtained. Based on Table 3, it can be concluded that the noise variance in the state transition process of the between-receiver phase-to-phase IFB is about 1 × 10^−10^–5 × 10^−10^ m^2^/s, and that of the between-receiver code-to-phase IFB is approximately 1 × 10^−6^–5 × 10^−6^ m^2^/s. These statistical results will be treated as empirical reference values for the following data processing.

### 4.2. Time Synchronization Precision and Frequency Stability Analysis

According to the above data processing strategies summarized in Table 2, the BDS code and phase observations collected from the zero and short baselines were processed to achieve the ambiguity fixed solution of the between-receiver clock difference and other parameters. As mentioned before, to suppress the influence of measurement noise, we simply picked the observations of the highest-elevation satellite, to establish the equation in each epoch, and the Kalman filter was employed to estimate the unknown parameters. When the picked satellite changed, the Kalman filter was initialized in order to check the consistency of different satellite solutions and to ensure the reliability of the estimation results. As is known, by using the Kalman filter for parameter estimation, the initial status of the estimated parameters shall be configured. Therefore, the initial value, initial variance, and noise variance of the state transition process of the estimated parameters were given, as shown in Table 4. It should be noted that the between-receiver IFB was constrained at the initial epoch, and its state change process was described by the random walk model. After the floating-point solution of the phase ambiguity was obtained, the LAMBDA method was used to search for the integer options of the phase ambiguity. Then, the optimal and suboptimal integer solutions were tested using the ratio testing function. If the ratio value was greater than the threshold value (set as 2 in this research), it was considered that the phase ambiguity was fixed correctly. Once the integer ambiguity was determined, it was submitted into the observation equations to recalculate the parameters.

**Figure 3:**
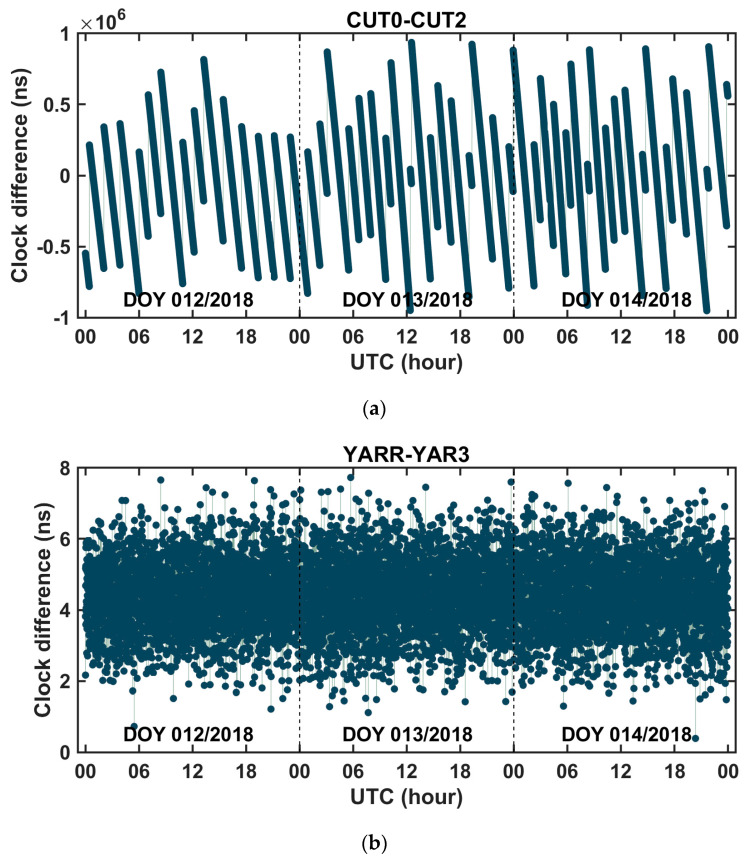
Time series of between-receiver clock difference with single-difference ambiguity resolution on days 12–14 in 2018 at time links (**a**) CUT0–CUT2 and (**b**) YARR–YAR3.

**Figure 4:**
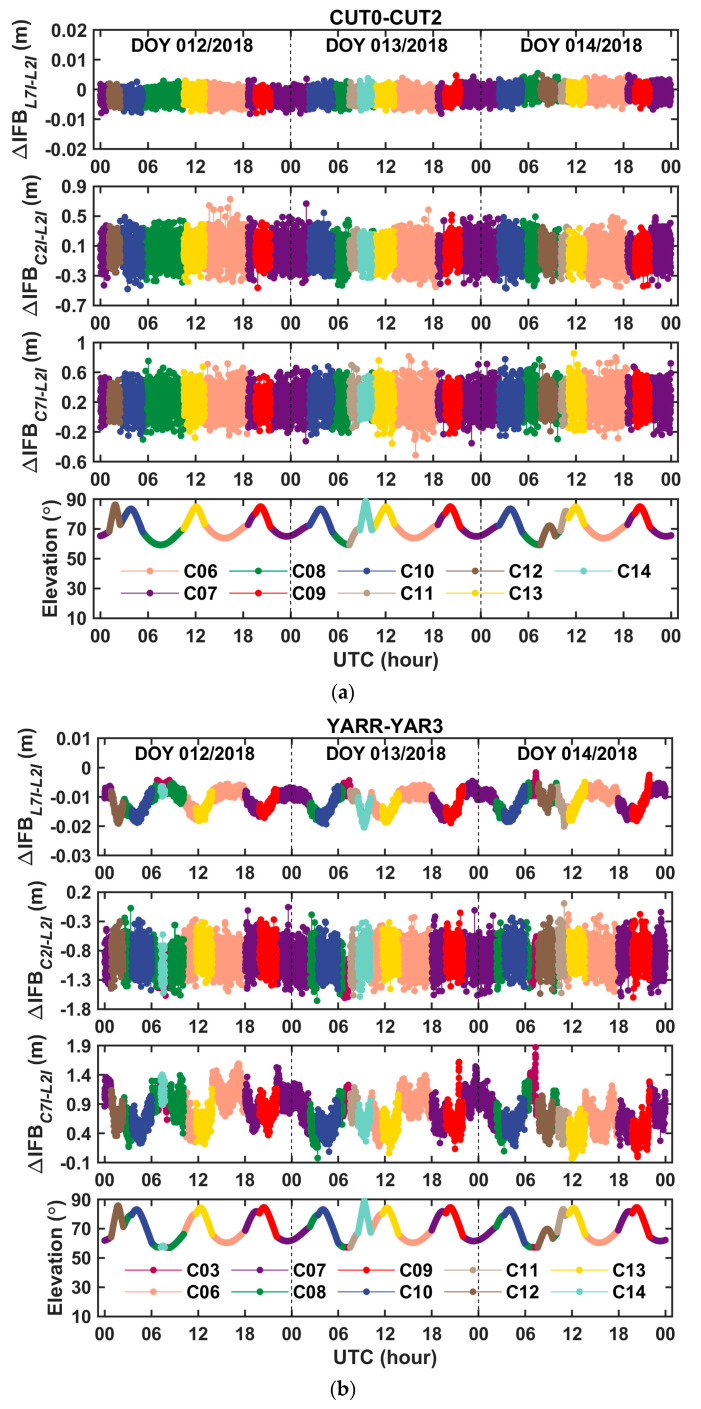
Time series of between-receiver IFBs with single-difference ambiguity resolution on days 12–14 in 2018 for (**a**) the zero baseline CUT0–CUT2 and (**b**) the short baseline YARR–YAR3.

**Figure 5:**
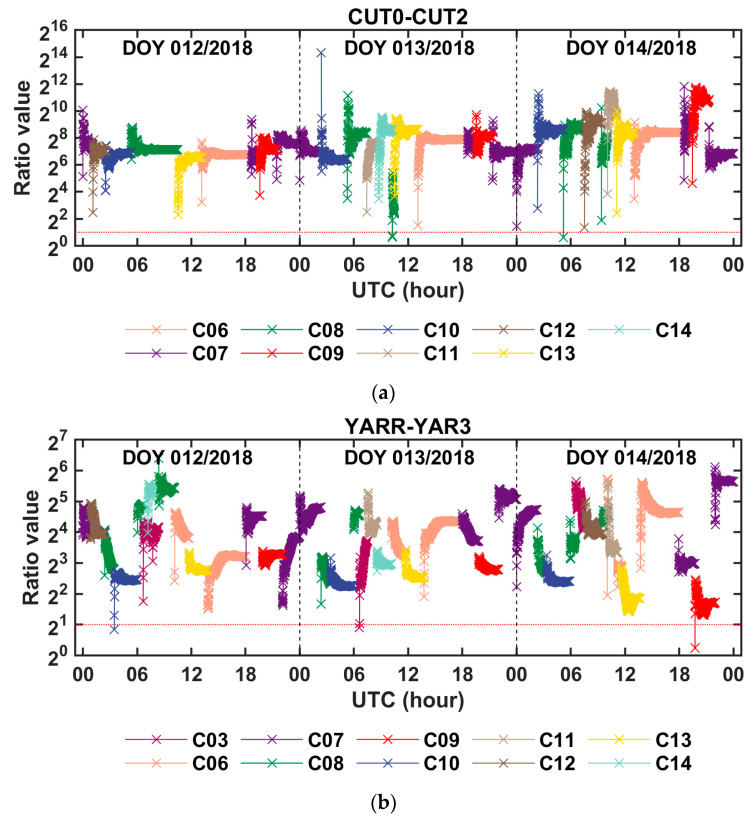
Ratio test value for the integer ambiguity acceptance in each epoch of (**a**) the zero baseline CUT0–CUT2 and (**b**) the short baseline YARR–YAR3.

**Figure 6:**
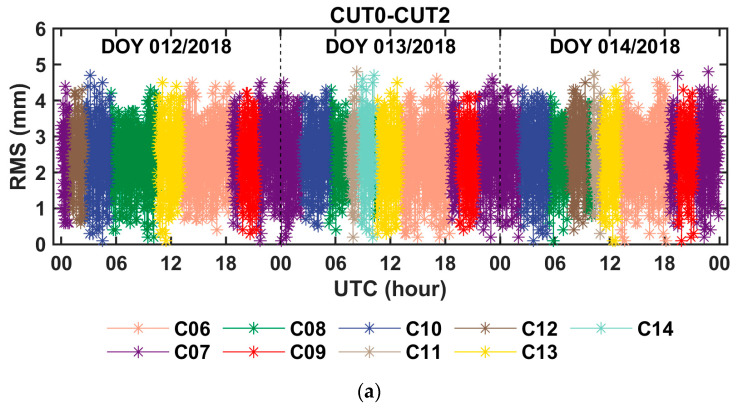

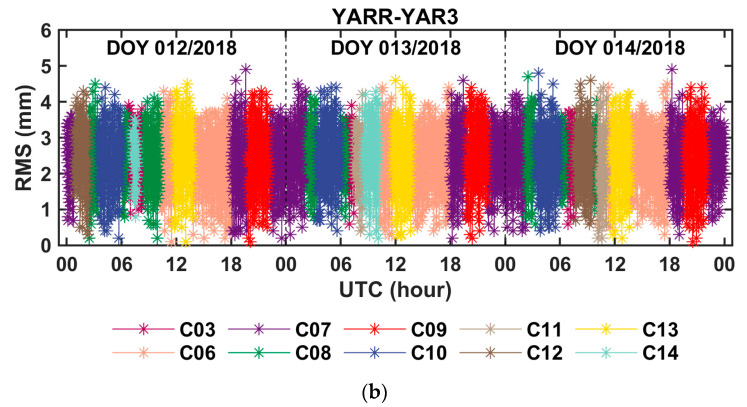
Root mean square (RMS) value of the between-receiver clock difference with single-difference ambiguity resolution: (**a**) the zero baseline CUT0–CUT2 and (**b**) the short baseline YARR–YAR3.

Three-day time series of the fixed solutions of the between-receiver clock difference and the between-receiver IFBs, that were derived from the BDS phase and code observations of the zero baseline CUT0–CUT2 and the short baseline YARR–YAR3, are shown in Figure 3 and Figure 4, respectively. Corresponding to Figure 3 and Figure 4, Figure 5 shows the ratio test value for the ambiguity fixation in each epoch. It can be seen that, when the threshold value of the ratio test was set to 2, in most cases, the single-difference ambiguity could be fixed successfully in a single epoch, and in a few cases, several epochs were needed. Table 5 presents the statistical results of the time-to-first-fix (TTFF) of the single-difference ambiguity determination for all satellites. From the table, we can conclude that the averaged epoch number needed for single-difference ambiguity fixation was fewer than 2 epochs, so this method can quickly achieve the fixation of the single-difference ambiguity. From Figure 4, it can also be seen that the fixed solution of the between-receiver IFBs obtained from different satellites was consistent, which further proved the reliability of the ambiguity fixed solution. At the same time, the fluctuation in the between-receiver phase-to-phase IFB estimation of the short baseline was greater than that of the zero baseline. We think that this was mainly caused by multipath effects. Once the single-difference ambiguity was fixed correctly, the carrier phase turned to be the unambiguous pseudorange with high precision. Since the measurement noise of the carrier phase observation is on the millimeter order, the precision of the fixed solution of the between-receiver clock difference was at the level of several millimeters. Considering that when the satellite elevation is high, the error residual such as the phase multipath effect is about a few millimeters, the precision of the obtained clock difference is expected to be millimeter-level. The root mean square (RMS) values of the fixed solution of the between-receiver clock difference were calculated as an indicator to evaluate the precision of the estimated parameter. The results are shown in Figure 6. The figure reveals that the RMS of the relative clock offset estimation with the single-difference ambiguity solved was about 3 mm, so its equivalent time synchronization precision can reach about ten picoseconds. According to the formal errors, and taking into account the influence of the error residuals, we can conclude that time synchronization on tens of picoseconds level can be achieved using the uncombined carrier phases of the zero and short baselines. In addition, due to the ambiguity of each satellite being solely fixed and only the carrier phase observation being used to calculate the between-receiver clock difference, the two epoch solutions that connected with the day were continuous without jumping, eliminating the problem of “day boundary discontinuity” in the time transfer.

**Figure 7:**
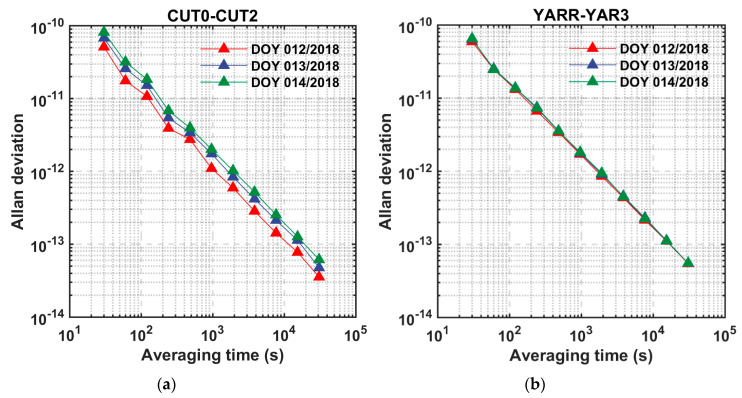
Allan deviation for the fixed solution of the clock difference on days 12–14 in 2018 at time links (**a**) CUT0–CUT2 and (**b**) YARR–YAR3.

Furthermore, the frequency stability of the time link is a crucial indicator of the time and frequency transfer. In order to further analyze the stability of the receiver clock offset from the perspective of frequency stability, the Allan variance of the fixed solution of the relative clock offset at different time scales was statistically analyzed [32]. Since there was a significant drift in the clock difference estimation of the zero baseline CUT0–CUT2, a linear model was used to remove the drift on arc-by-arc basis. After that, the Allan deviations were calculated. As shown in Figure 7, the average frequency stability at 30,000 s is better than 5 × 10^−14^ of the short baseline YARR–YAR3, and that of the zero baseline CUT0–CUT2 is also around 5 × 10^−14^. The stability between days is basically the same, which demonstrates the stability and repeatability of this method for time-frequency synchronization.

### 4.3. Comparison with PPP Solution

**Figure 8:**
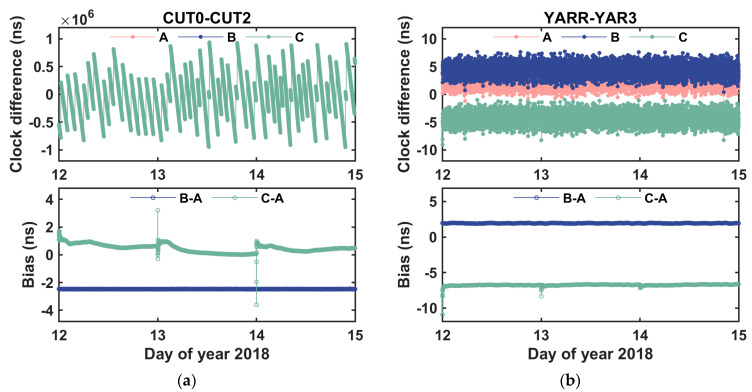
Between-receiver clock differences derived from three different solutions at time links (**a**) CUT0–CUT2 and (**b**) YARR–YAR3.

In order to confirm the validity of the proposed method, the results derived from our proposed method were compared with the PPP solution. The online PPP tool of Canadian Spatial Reference System (CSRS-PPP) was employed to estimate the receiver clock offset, using the ionosphere-free code and phase combinations in a station-by-station manner. Then, the clock difference between two receivers were formed. The clock differences derived from three solutions, i.e., our method using GPS observations (solution A), our method using BDS observations (solution B), and PPP method using GPS ionosphere-free combinations (solution C), were shown in Figure 8.

It can be seen that solution A and solution B have a good agreement. However, a systematic bias was observed. This bias is the inter-system frequency bias between GPS L1C carrier phase and BDS L2I carrier phase. From Table 6, we can see that the bias is about −2.47 ns for the baseline CUT0–CUT2, and it is about 1.94 ns for the baseline YARR–YAR3. The standard deviations of the biases are about 0.006 ns and 0.03 ns, respectively, indicating that the proposed method has good stability. In the meantime, a systematic deviation between solution A and solution C was also observed. Since the PPP method has a convergence period, the first hour data in the result of PPP solution were eliminated. Then, the differences between solution A and solution C on days 12–14 in 2018 were statistically summarized in Table 6. The daily average deviation of the baseline CUT0–CUT2 varies from 0.2 ns to 0.7 ns for three consecutive days. Whereas, for the baseline YARR–YAR3, the mean value of the deviations between solution A and solution C is stable at around −6.7 ns. We think that this deviation is mainly caused by the receiver hardware delay on GPS L1C carrier phase and the receiver hardware delay on GPS ionosphere-free code combination formed by GPS C1C and C2W. Hence, a systematic bias ranging from sub-nanosecond to several nanoseconds was found between our method and PPP method. This kind bias is mainly induced by the receiver hardware delay of carrier phase and receiver hardware delay of ionosphere-free code combination, and it depends on the type of receiver.

## 5. Summary and Conclusions

In this contribution, we investigated the mathematical principle of time and frequency synchronization, based on the uncombined GNSS carrier phase observations. In view of the issue that the precision and efficiency of time and frequency transfer being easily affected by the receiver hardware inter-frequency bias (IFB), a method that took the priori constraints of the between-receiver IFB and its random variation characteristics into account, was proposed, to determine the relative clock offset between two receivers with the single-difference ambiguity resolution. First, we analyzed and modeled the temporal variation characteristics of between-receiver IFBs. Then, considering the priori constraints and the state change model of the between-receiver IFBs, the ambiguity fixed solution of the between-receiver clock difference was achieved, and the between-receiver IFBs were calibrated based on the zero/short baseline mode.

The validity and reliability of the proposed method were analyzed by using the BDS/GPS dual-frequency carrier phase and pseudorange observations, that were dedicatedly collected from the zero and short baselines on several consecutive days. The experimental results show that the between-receiver single-difference ambiguity can be fixed successfully within an average of fewer than 2 epochs (interval 30 s). After the integer solution of the single-difference ambiguity was determined correctly, the between-receiver clock difference with a millimeter precision was obtained by using only the uncombined carrier phase observations. Additionally, the fixed solution of the between-receiver IFBs was obtained. Three-day time series of the fixed solution of between-receiver clock difference indicates that this method can achieve the time synchronization on tens of picoseconds level precision, and it has good stability. Since the single-difference ambiguity is resolved successfully, the daily boundary of time transfer is also continuous. Besides, for the tested short baseline, the average stability of 30,000 s for the proposed CP time and frequency method is better than 5 × 10^−14^. Furthermore, comparative experiments show that, for the type of receiver tested, the agreement between our approach and the PPP method is between −6.7 ns and 0.2 ns, and this deviation is mainly caused by the receiver hardware bias of carrier phase and receiver hardware bias of ionosphere-free code combination.

## Figures and Tables

**Table 1:** Details of Global Navigation Satellite System (GNSS) data sets used in the experiment of time synchronization.

Station	Receiver	Antenna	Observation	Time Period
CUT0	TRIMBLE NETR9	TRM59800.00	BDS: C2I, C7I, L2I, L7I GPS: C1C, C2W, L1C, L2W	2018, days 12–14
CUT2	TRIMBLE NETR9			
YARR	SEPT POLARX5	LEIAT504		
YAR3	SEPT POLARX5	LEIAR25		

**Table 2:** Data processing strategies.

Items	Strategies
Between-receiver clock difference	Estimated, linear model
Between-receiver phase-to-phase inter-frequency bias (IFB)	Estimated, random walk model
Between-receiver code-to-phase IFB	Estimated, random walk model
Ambiguities	Estimated, constants
Satellite orbit	Fixed by Helmholtz-Centre Potsdam (GFZ) precise orbit products
Station coordinates	Fixed by known position (ITRF 2014)
Phase wind-up effect	Corrected
Antenna offset	International GNSS Service (IGS) values
Observations	Single-difference code and phase observations
Observations weight	Elevation weight
Estimator	Kalman filter

**Table 3:** State process noise variance of the between-receiver IFBs.

Baseline	DOY (2018)	ΔIFB*_L7I-L2_*			ΔIFB*_C2I-L2I_*			ΔIFB*_C7I-L2I_*		
		*e*	*σ*	*w*	*e*	*σ*	*w*	*e*	*σ*	*w*
CUT0–CUT2	12	0.00	0.02	1.21 × 10^−10^	0.00	1.80	9.73 × 10^−7^	0.01	1.85	1.02 × 10^−6^
	13	0.00	0.02	1.27 × 10^−10^	0.00	1.78	9.56 × 10^−7^	0.02	1.93	1.12 × 10^−6^
	14	0.00	0.02	1.20 × 10^−10^	0.02	1.81	9.87 × 10^−7^	−0.01	1.89	1.07 × 10^−6^
YARR–YAR3	12	0.00	0.04	3.77 × 10^−10^	0.09	2.55	1.95 × 10^−6^	−0.01	2.91	2.53 × 10^−6^
	13	0.00	0.04	4.37 × 10^−10^	0.14	2.54	1.94 × 10^−6^	−0.13	2.81	2.36 × 10^−6^
	14	0.00	0.04	5.14 × 10^−10^	0.07	2.65	2.11 × 10^−6^	−0.27	2.87	2.46 × 10^−6^

**Table 4:** Parameter status setting.

Parameter	Initial Value	Initial Variance	Process Noise Variance
Between-receiver clock difference	Pseudorange estimation	360,000 m^2^	0.5 m^2^/s
Velocity of clock difference	Pseudorange estimation	360,000 m^2^/s^2^	0.03 m^2^/s^2^
Between-receiver phase-to-phase IFB	0 m	0.16 m^2^	1 × 10^−9^ m^2^/s
Between-receiver code-to-phase IFB	0 m	36 m^2^	1 × 10^−5^ m^2^/s
Single-difference ambiguity	Pseudorange estimation	1.0 × 10^12^ cycle^2^	0 cycle^2^/s

**Table 5:** Time-to-first-fix (TTFF) of the between-receiver single-difference ambiguity.

Baseline	DOY (2018)	Arc Count	Total TTFF (30 s)	Average TTFF (30 s)
CUT0–CUT2	12	9	9	1.0
	13	11	13	1.2
	14	11	12	1.1
YARR–YAR3	12	15	16	1.1
	13	13	15	1.2
	14	15	16	1.1

**Table 6:** Statistics of differences between different solutions.

Time link	DOY (2018)	Difference between Solutions A and B		Difference between Solutions A and C	
		Mean (ns)	STD (ns)	Mean (ns)	STD (ns)
CUT0–CUT2	12	−2.475	0.006	0.700	0.160
	13	−2.473	0.006	0.228	0.272
	14	−2.474	0.006	0.415	0.116
YARR–YAR3	12	1.937	0.028	−6.787	0.041
	13	1.938	0.026	−6.732	0.052
	14	1.935	0.028	−6.732	0.057
